# Mechanically durable, superoleophobic coatings prepared by layer-by-layer technique for anti-smudge and oil-water separation

**DOI:** 10.1038/srep08701

**Published:** 2015-03-03

**Authors:** Philip S. Brown, Bharat Bhushan

**Affiliations:** 1 Nanoprobe Laboratory for Bio- & Nanotechnology and Biomimetics (NLBB), The Ohio State University, 201 W. 19^th^ Avenue, Columbus, OH 43210-1142, USA

## Abstract

Superoleophobic surfaces are of interest for anti-fouling, self-cleaning, anti-smudge, low-drag, anti-fog, and oil-water separation applications. Current bioinspired surfaces are of limited use due to a lack of mechanical durability. A so-called layer-by-layer approach, involving charged species with electrostatic interactions between layers, can provide the flexibility needed to improve adhesion to the substrate while providing a low surface tension coating at the air interface. In this work, a polyelectrolyte binder, SiO_2_ nanoparticles, and a fluorosurfactant are spray deposited separately to create a durable, superoleophobic coating. Polydiallyldimethylammonium chloride (PDDA) polyelectrolyte was complexed with a fluorosurfactant layer (FL), which provides oil repellency while being hydrophilic. This oleophobic/superhydrophilic behavior was enhanced through the use of roughening with SiO_2_ particles resulting in a superoleophobic coating with hexadecane contact angles exceeding 155° and tilt angles of less than 4°. The coating is also superhydrophilic, which is desirable for oil-water separation applications. The durability of these coatings was examined through the use of micro- and macrowear experiments. These coatings currently display characteristics of transparency. Fabrication of these coatings via the layer-by-layer technique results in superoleophobic surfaces displaying improved durability compared to existing work where either the durability or the oil-repellency is compromised.

Bioinspired, superoleophobic surfaces (oil contact angles greater than 150°, contact angle hysteresis or tilt angle less than 4°) have properties that make them ideal for anti-fouling, self-cleaning, anti-smudge and low-drag applications^1^,^2^,^3^,^4^,^5^,^6^,^7^,^8^,^9^. Such surfaces could find use in the building, transportation, and electronics industries^6^.

A liquid droplet in contact with a flat, solid surface does so with a contact angle characteristic of both the liquid and the solid in question according to the Young's equation^10^
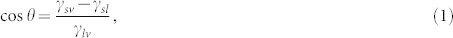
where *γ_sv_*, *γ_sl_*, and *γ_lv_*are the solid-vapor, solid-liquid, and liquid-vapor surface tensions respectively, and *θ* is the contact angle of the droplet. The solid-liquid interactions can be enhanced through the use of surface roughness. Increasing the surface area in contact with the droplet leads to amplification of the solid-liquid interaction, repellent surfaces therefore become more repellent or liquids spread further on non-repellent surfaces. This assumes that the surface is fully wetted by the liquid and is known as the Wenzel regime^11^. Another scenario when considering droplets on rough surfaces is that air pockets become trapped between the surface and the liquid resulting in a composite interface known as the Cassie-Baxter regime^12^. In this case the surface typically becomes more repellent since the droplet is resting partially on air pockets. Therefore, it is possible for non-repellent surfaces to become repellent through an increase in roughness and the formation of a composite air/solid interface.

Many examples of superoleophobic surfaces exist; these typically involve a fluorinated component to provide the low surface tension^13^ and a roughness component to enhance the liquid-solid interactions. An early example used rough, oxidized aluminium surfaces immersed in fluorinated monoalkylphosphates^14^; resulting in oil and water contact angles of 150°. A fluorinated polyhedral oligomeric silsesquioxane has been used in various scenarios - including electrospinning and coating re-entrant structures - to create a superoleophobic coating^2^,^15^. However, a superoleophobic surface with good mechanical durability typically has not been demonstrated. Yang et al. spray coated copper perfluorooctanoic acid to create a rough, superoleophobic surface however it was found to be susceptible to scratching^16^. Conversely, chemical vapor deposition of a fluorosilane on an SiO_2_ aerogel is found to result in a durable coating, however the oleophobicity is poor with high contact angle hysteresis values for mineral oil^17^. Poor durability can be due to the “one-pot” technique typically utilized in these studies, where the low surface tension material required for oleophobicity is distributed throughout the coating, compromising adhesion to the substrate^8^.

These superoleophobic composite surfaces are typically superhydrophobic as well; a surface that repels oil usually also repels water since water has a higher surface tension (Eq. 1). For oil-water separation applications, it is of interest to have a surface that can repel oils while being wet by water. This behavior is typically achieved through surface reorganization. One common example is a polyelectrolyte-fluorosurfactant complex surface^18^,^19^. Such a surface results in a finite contact angle for oils due to the low surface energy fluorine tail groups of the surfactant. In contrast, water is able to penetrate through the fluorinated layer down to the more hydrophilic sub-layer where the surfactant head is complexed with the oppositely charged polyelectrolyte.

Early work on polyelectrolyte-fluorosurfactant complex coatings with a view to providing oleophobicity was carried out by Antonietti et al.^20^. Such films were found to have a higher contact angle for hexadecane (surface tension 27 mN m^−1^) than for glycerol (surface tension 64 mN m^−1^). Sawada et al.^21^ investigated fluorinated silanes which contained hydrophilic groups and found that while the oil contact angle remained high, the water contact angle decreased from 80° to 0° over the course of 25 minutes; hence they were named “flip-flop” surfaces. The same group later went onto report a similar behavior on fluorinated oligomer-polymer hybrids^22^ and oligomer-calcium silicide nanocomposites^23^. Other investigators found that fluorosurfactant complexed with a pulsed plasma deposited polymer coating resulted in similar behavior of high oil contact angles and low water contact angles; such behavior was attributed to surface reconstruction upon addition of water^24^,^25^. While these studies provided the properties desired, the oleophobicity/hydrophilicity achieved was poor.

In an attempt to improve the oleophobicity/hydrophilicity, roughness was introduced to enhance the liquid-surface interactions. Yang et al.^26^ mixed polymer, fluorosurfactant and nanoparticles together to result in a superoleophobic, superhydrophilic coating. However, the time taken for water to penetrate down to the hydrophilic portions of the coating was found to be too long (ca. 10 min); thus the coating was initially superhydrophobic unless treated with air plasma, limiting the possible applications. In addition, the mechanical durability of the coating was not investigated.

A coating that repels oil but is wet by water would have numerous applications. In addition to being self-cleaning^27^,^28^,^29^, anti-fouling^30^,^31^, and anti-smudge - like oil and water repellent coatings - these coatings could also be used for anti-fogging^27^,^32^,^33^ or oil-water separation^34^,^35^ applications. In the case of the latter, hydrophilic/oleophobic is thought to be more favorable than hydrophobic/oleophilic for separation of oil and water since water is more dense than oils and tends to sink to the bottom of a mixture and oleophilic surfaces quickly become fouled by oils requiring cleaning or replacement^36^,^37^.

Typical examples of oleophobic/hydrophilic surfaces involve the use of a polyelectrolyte and a fluorosurfactant mixed together in a one-pot technique^26^,^35^. Another deposition method utilizing similar interactions between charged components is known as the “layer-by-layer” technique, where components are kept separate and deposited individually. Layers of oppositely charged species are deposited one after another to create a multi-layer coating bound together through electrostatic interactions. Many different charged species can be utilized when creating the layered coating and therefore the technique is highly flexible and has been used in a variety of applications, Table 1. Due to the use of water-soluble polymers, layer-by-layer coatings are typically hydrophilic and oleophilic. However, the final polyelectrolyte layer in the multi-layer coating could be further functionalized with an oppositely charged surfactant. This would enable the use of fluorinated material solely at the air interface; therefore ensuring that the adhesion of the coating to the substrate would not be compromised by low surface tension material.

In the present study, a superoleophobic/superhydrophilic coating is developed via a polyelectrolyte-fluorosurfactant complex and SiO_2_ nanoparticles to increase the roughness. An adapted layer-by-layer technique involving the deposition of separate layers was used to ensure good adhesion between the substrate and good coverage of the functional layer (fluorosurfactant) at the air interface. The coating was found to be mechanically durable, with properties of transparency. Such a novel coating could be used for anti-fouling (where superoleophobicity, superhydrophilicity and nanostructuring are all of importance for anti-biofouling), self-cleaning, anti-smudge, low-drag, anti-fog, and oil-water separation applications.

## Experimental details

**Figure 1:**
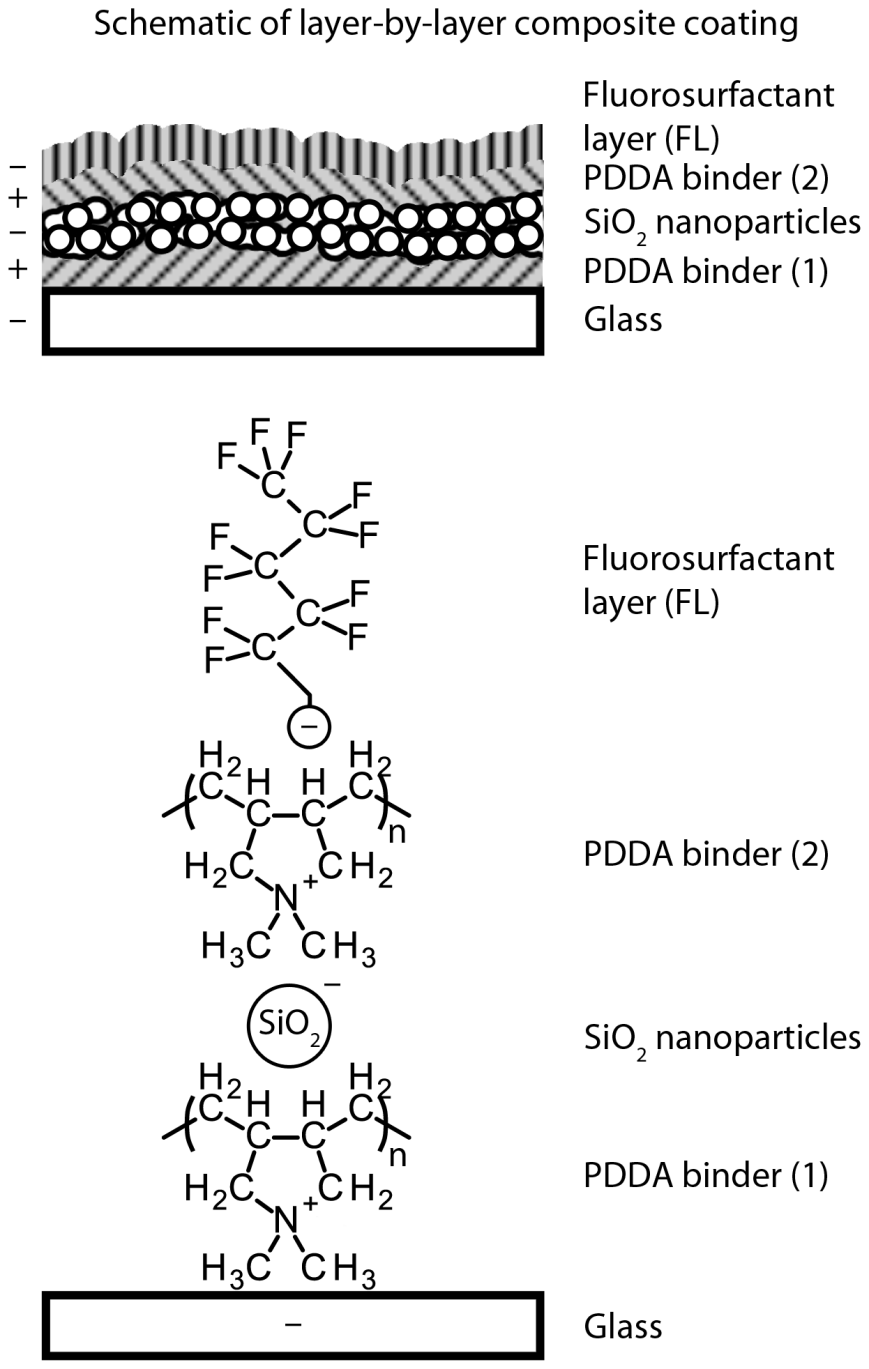
Schematic of layer-by-layer composite coating. Each layer is spray coated separately. Also shown are the chemical composition and charge of each layer.

The coating described in this paper comprises four layers, deposited separately, each of which aids the creation of a mechanically durable, superoleophobic coating. Fig. 1 shows the final coating composition as well as the chemical structures of the individual components. PDDA was chosen as the polymer base layer as it has been shown to adhere well to glass substrates^38^,^39^ and SiO_2_ nanoparticles with known wear resistance^40^. Untreated, hydrophilic SiO_2_ nanoparticles were used to enhance the roughness of the coating and to ensure good adhesion to the positively charged polymer layers thanks to a negative surface charge from ionized surface silanol groups. In addition, SiO_2_ nanoparticles are known to have high hardness^41^, which will aid in the creation of a mechanically durable coating^42^. Particles of 7 nm in diameter were selected in the hope that coating transparency would be maintained. The fluorosurfactant was selected to provide oil-repellency because of its low surface tension tail and ability to complex to a positively charged polyelectrolyte from its high surface tension head group.

### Samples

Glass slides (Fisher Scientific) cut to dimensions of 25 by 10 mm were used as substrates. Polydiallyldimethylammonium chloride (PDDA, MW 100,000–200,000, Sigma Aldrich) was dissolved in distilled water (DS Waters of America Inc.) at various concentrations. Silica nanoparticles (NP, 7 nm diameter, Aerosil 380, Evonik Industries) were dispersed in acetone (Fisher Scientific Inc.) using an ultrasonic homogenizer (Branson Sonifier 450 A, 20 kHz frequency at 35% amplitude) at various concentrations. The fluorosurfactant solution (FL, Capstone FS-50, DuPont) was diluted with ethanol (Decon Labs Inc) so that the overall fluorosurfactant concentration was 45 mg mL^−1^. Coatings were deposited via spray gun (Paasche) operated with compressed air at 210 kPa. The gun was held 10 cm from the glass slide at all times. For the final composite coating, four spray depositions were used. First, PDDA solution (52 mg mL^−1^, 2 mL) was spray coated and any excess was removed from the surface via bursts of compressed air from the spray gun. Second, the SiO_2_ NP solution (various concentrations, 3 mL) was spray coated. Third, a second PDDA layer was deposited (various concentrations, 1 mL). After this, the samples were transferred to an oven operating at 140°C for 1 h. Finally, the fluorosurfactant solution (1 mL) was spray coated and the samples were allowed to dry in air.

### Contact angle and tilt angle

For contact angle data, droplets of 5 μL volume were deposited onto samples using a standard automated goniometer (Model 290, Ramé-Hart Inc.) and the resulting image of the liquid–air interface analyzed with DROPimage software. Tilt angles were measured by inclining the surface until a 5 μL droplet rolled off. Contact angle hysteresis was measured by tilting the substrate until the droplet was observed to move and the advancing and receding angles were recorded. These numbers were found to be comparable to the tilt angles and are not reported. All angles were averaged over at least five measurements on different areas of a sample. Oils tested include n-hexadecane (99%, Alfa Aesar), n-tetradecane (≥99%, Sigma Aldrich), n-dodecane (≥99%, Sigma Aldrich), n-decane (≥99%, Sigma Aldrich), and n-octane (≥99%, Sigma Aldrich).

### Surface topography and coating thickness

The surface topography of each sample was determined using a D3000 Atomic Force Microscopy (AFM) with a Nanoscope IV controller (Bruker Instruments). A Si, n-type (Si_3_N_4_) tip with an Al coating (resonant frequency f = 66 kHz, spring constant k = 3 N m^−1^, AppNano) operating in tapping mode was used. The scanning area was 1 × 1 μm^2^ to determine roughness on the nanoscale. Root mean square roughness (RMS) and Peak-to-Valley (P–V) distance values were obtained.

Coating thickness of each individual layer and the composite coating was measured with a step technique. One half of the substrate was covered with a glass slide using double-sided sticky tape before coating and then removed after the coating procedure resulting in a step. An area including the step was imaged by AFM to obtain the coating thickness.

### Wear experiments

The mechanical durability of the surfaces was examined through wear experiments using an AFM and a ball-on-flat tribometer^43^. An established AFM micro-wear procedure was performed with a commercial AFM (D3000, Nanoscope IV controller, Bruker Instruments). Surfaces were worn using a borosilicate ball with radius 15 μm mounted on a rectangular cantilever with nominal spring constant of 7.4 N m^−1^ (resonant frequency f = 150 kHz, All-In-One). Areas of 50 × 50 μm^2^ were worn for 1 cycle at a load of 10 μN so as to be later imaged within the scanning limits of the AFM. To analyze the change in morphology of the surface before and after the wear experiment, height scans of 100 × 100 μm^2^ in area were obtained using a Si, n-type (Si_3_N_4_) tip with an Al coating (resonant frequency f = 66 kHz, k = 3 N m^−1^, AppNano) operating in tapping mode. Root mean square roughness (RMS) values before and after wear experiments were obtained.

Macrowear experiments were performed with an established procedure of using a ball-on-flat tribometer^42^. A sapphire ball of 3 mm diameter was fixed in a stationary holder. A load of 10 mN was applied normal to the surface, and the tribometer was put into reciprocating motion. Stroke length was 6 mm with an average linear speed of 1 mm s^−1^. Surfaces were imaged before and after the tribometer wear experiment using an optical microscope with a CCD camera (Nikon Optihot-2) to examine any changes^41^.

Contact pressures for both AFM and tribometer wear experiments were calculated based on Hertz analysis^42^. The elastic modulus of PDDA, 0.16 GPa^44^, was used to estimate the elastic modulus of the composite coating, and a Poisson's ratio of 0.5 was used (estimated). The elastic modulus of final coating is expected to be higher, so an underestimated pressure will be obtained with the selected modulus. The elastic modulus of 70 GPa and Poisson's ratio of 0.2 were used for the borosilicate ball used in the microscale wear experiments^45^. The elastic modulus of 390 GPa and Poisson's ratio of 0.23 were used for sapphire ball used in the macroscale wear experiments^46^. The mean contact pressures were calculated as 4.87 MPa and 2.26 MPa for the AFM (micro) and ball-on-flat tribometer (macro) experiments respectively. Microscale wear experiments were performed for 1 cycle while macroscale wear experiments were performed for 100 cycles. Therefore, the macroscale wear experiments can cause a relatively high degree of damage to the coating even though the mean contact pressures are comparable to the microscale technique.

### Anti-smudge experiment

The anti-smudge characteristics of the surfaces were examined using an experimental setup previously reported^8^,^47^. Coatings were contaminated with silicon carbide (SiC, Sigma Aldrich) in a glass chamber (0.3 m diameter and 0.6 m high) by blowing 1 g of SiC powder onto a sample for 10 s at 300 kPa and allowing it to settle for 30 min. The contaminated sample was then secured on a stage and a hexadecane-impregnated microfiber wiping cloth was glued to a horizontal glass rod (radius 0.5 mm) fixed on a cantilever above the sample. As the cloth was brought in contact with the sample, the microfiber cloth was set to rub the contaminated sample under a load of 5 g for 1.5 cm at a speed of about 0.2 mm s^−1^. Images were taken using an optical microscope with a CCD camera (Nikon, Optihot-2). The removal and transfer of nanoparticles by the cloth was compared before and after tests.

### Oil–water separation experiment

The superoleophobic/superhydrophilic nature of the coatings was tested with an oil–water separation experiment. The composite coating was deposited on stainless steel mesh (#400), which was cleaned with acetone and 2-propanol (Fisher Scientific) until it was found to be hydrophilic. The coated mesh was then placed on top of a beaker. An agitated mixture of hexadecane and water was then poured onto the coated mesh. In a separate experiment, the mesh was inclined at an angle and the oil–water mixture was poured over the mesh. To improve contrast, Oil Red O and Blue 1 were used as oil and water dispersible dyes respectively. The use of dyes was not found to have any effect on the performance of the coating.

## Results and Discussion

The final, optimized composite coating comprised of four separate layers (total thickness ca. 630 nm) each spray coated individually, Fig. 1. The first layer comprises PDDA (thickness ca. 200 nm) and acts as an anchor layer to the glass substrate. The second layer contains SiO_2_ nanoparticles (NP, thickness ca. 350 nm) and acts as the roughness layer, enhancing the overall liquid-solid interactions. The third layer is a second polymer coating (PDDA (2), thickness ca. 50 nm), which helps to bind the nanoparticle layer and improves adhesion and mechanical durability. The final layer is the fluorosurfactant layer (FL, thickness ca. 30 nm), which complexes with the positively charged PDDA (2) layer and provides the oil-repellency. Spray coating of a separate fluorosurfactant layer ensures correct functionality at the air interface (superoleophobicity) without compromising the durability of bulk coating.

### Wettability of coated surfaces

**Figure 2:**
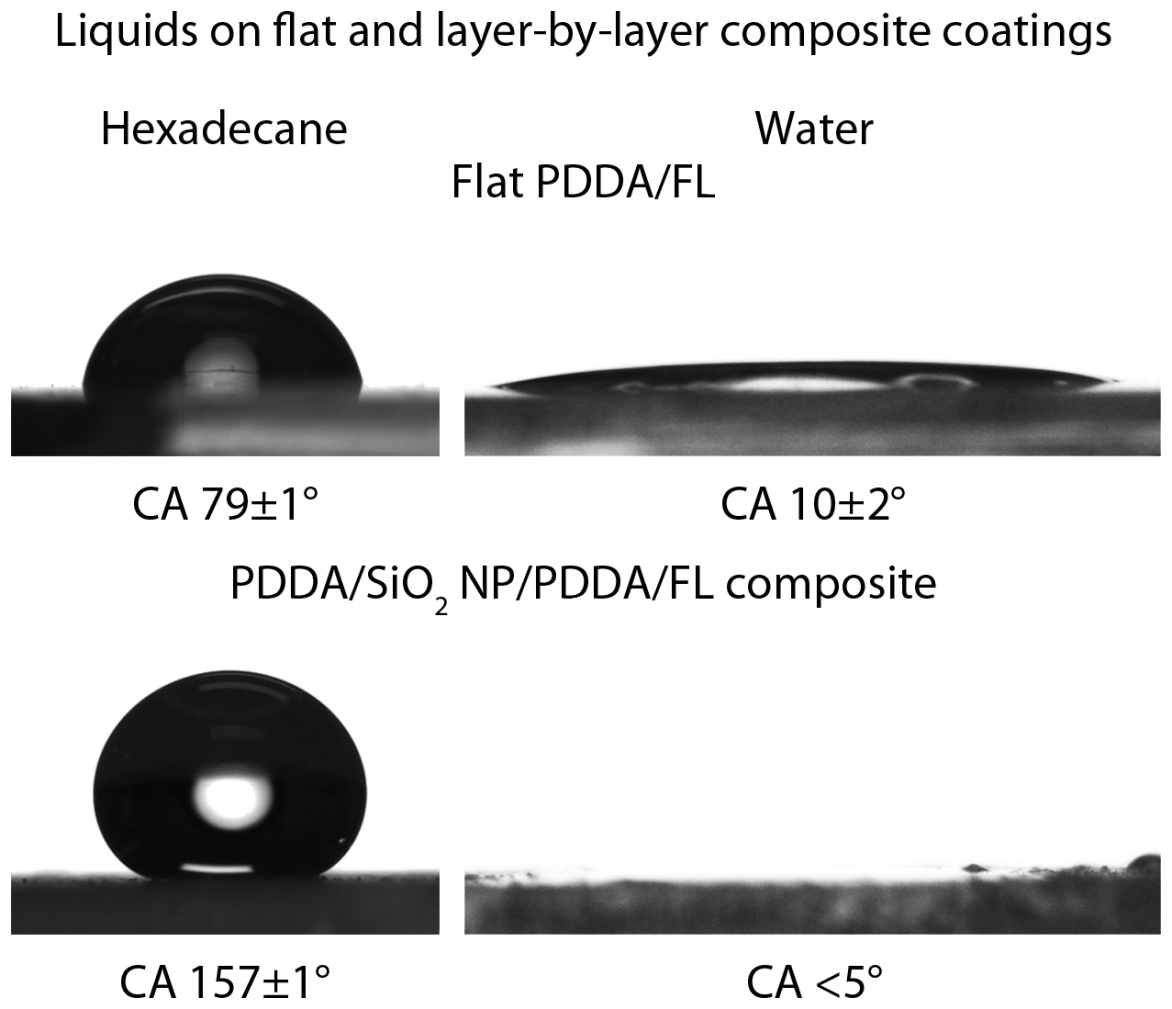
Hexadecane and water droplet deposited on flat and layer-by-layer composite coatings. Layer-by-layer composite coating concentrations: SiO_2_ NP 15 mg mL^−1^, PDDA (2) 8 mg mL^−1^.

Initially, two layer coatings of spray coated PDDA (52 mg mL^−1^, thickness ca. 200 nm) followed by spray coated FL (PDDA/FL) on glass substrates were created to determine the oil-repellency of the polyelectrolyte-fluorosurfactant complex. Fig. 2 shows representative pictures of the contact angles for water and hexadecane found on these coatings. Table 2 provides a summary of all contact angle data. The flat coating was found to result in finite contact angles for all oils tested. This is in contrast to bare glass and PDDA coatings, which were wet by all the oils (contact angle ca. 0°). While the PDDA/FL coating repels oils to some extent (finite contact angles), it retains the superhydrophilicity of the polyelectrolyte with a water contact angle of 10 ± 2°.

The behavior of oil-repellency, in addition to wetting by water, is due to the fluorosurfactant containing a low surface tension fluorinated tail and a high surface tension head group complexed with a hydrophilic polyelectrolyte, Fig. 1. During spray coating, the polar head group forms an electrostatic complex with the polyelectrolyte layer below and the fluorinated tails orient themselves at the air interface. Large oil molecules are trapped at this fluorinated interface while smaller water molecules can more easily penetrate down to the hydrophilic region where the surfactant head group complexes with the polyelectrolyte layer^35^,^48^. The result is a surface that repels oils but is wet by water.

**Figure 3:**
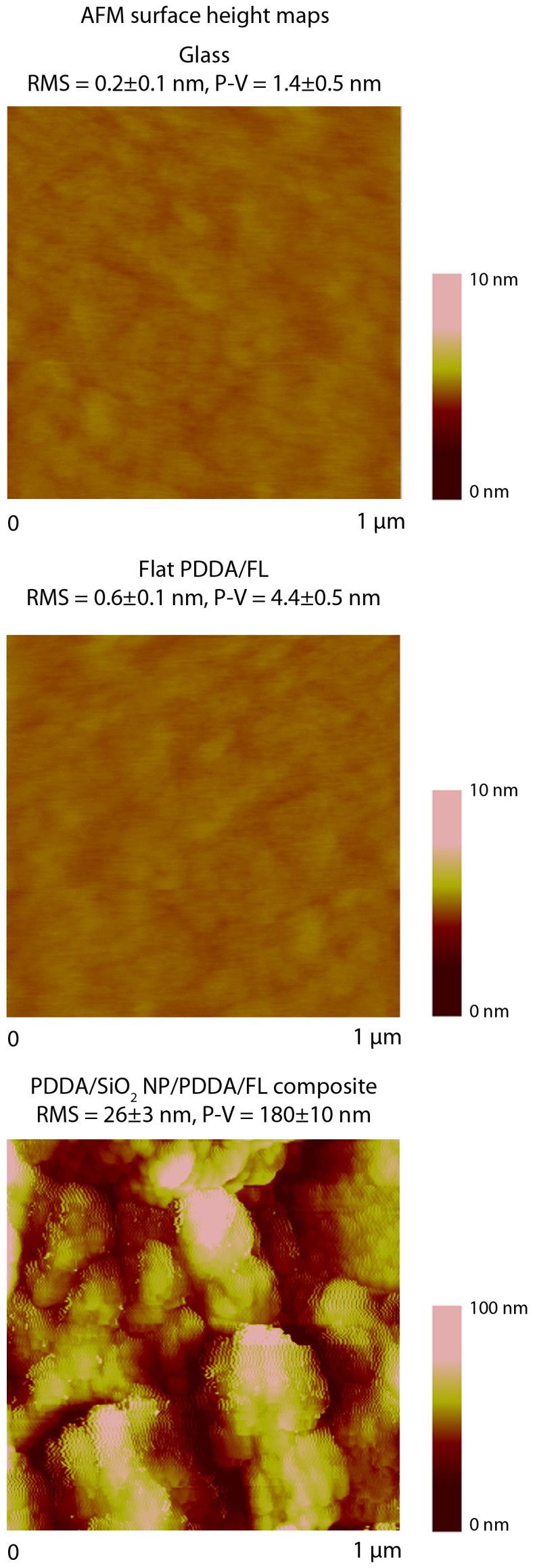
AFM surface height maps with RMS roughness values for glass, PDDA/FL, and layer-by-layer composite coating. Layer-by-layer composite coating concentrations: SiO_2_ NP 15 mg mL^−1^, PDDA (2) 8 mg mL^−1^.

To enhance the oil-repellency of the surface, roughness was introduced to the coating via spray deposition of a SiO_2_ nanoparticle layer (15 mg mL^−1^). Fig. 3 shows the AFM surface height maps and RMS values determined from several 1 × 1 μm^2^ scan areas of the various surfaces. This increase in roughness results in an increase in the oil contact angle (Fig. 2 and Table 2) due to the formation of a composite air/solid interface typically known as the Cassie-Baxter state. The addition of roughness also leads to a further decrease in the water contact angle due to water droplets being in the Wenzel state of wetting. Water droplets were found to immediately wet the surface in contrast to previous work where water penetration can take several minutes^26^. This is due to the fluorosurfactant only being present as a single layer at the air interface allowing water to wick down to hydrophilic polyelectrolyte layer beneath.

**Figure 4:**
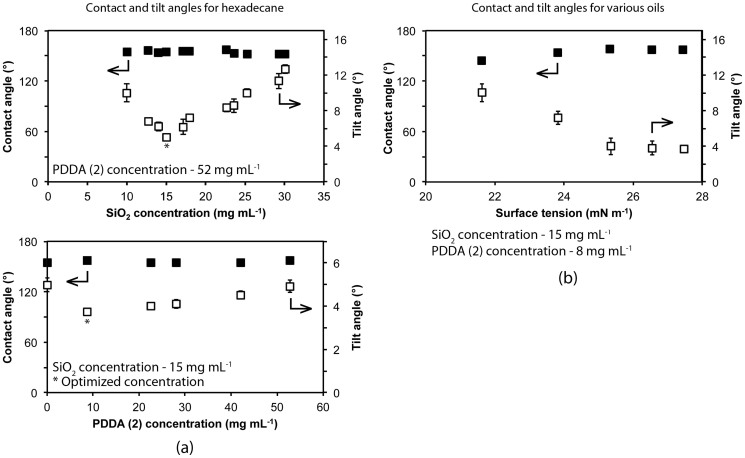
(a) Static contact angle and tilt angle for hexadecane as a function of SiO_2_ and PDDA (2) concentrations and (b) static contact angle and tilt angles for various liquids on the optimized surface as a function of liquid surface tension. Oils used: octane (21.14 mN m^−1^), decane (23.37 mN m^−1^), dodecane (25.35 mN m^−1^), tetradecane (26.13 mN m^−1^) and hexadecane (27.05 mN m^−1^).

To ensure optimal oil-repellency, the concentrations of the SiO_2_ NP and PDDA (2) coating solutions were varied and the hexadecane contact and tilt angles measured. The concentration of the PDDA (1) anchor layer was kept constant (52 mg mL^−1^) as this was found to have no effect on the oil-repellency. The data shown in Fig. 4a indicates that, for all NP concentrations studied, hexadecane contact angles remained at around 150° indicating that droplets were in the Cassie-Baxter regime on all coatings. It was found that at higher NP concentrations, the tilt angle for hexadecane remained fairly high. This is thought to be due to an increased number of particles increasing the solid contribution to the composite air/solid interface underneath the droplet (therefore decreasing the number of air pockets). Increasing the amount of surface in contact with the droplet will better pin the droplets in place leading to an increase in hysteresis^6^. By reducing the NP concentration, the hexadecane tilt angle is reduced due to an increase in the air contribution to the interface and a decrease in pinning. Below a concentration of 15 mg mL^−1^, the tilt angles begin to rise again. This is due to a decrease in the amount of trapped air between the surface and the liquid leading to a greater adhesive force between the two and a corresponding increase in hysteresis. The concentration of the second polymer layer (PDDA (2)) was also found to affect the oil repellency. At higher concentrations the layer is too thick and can reduce the effectiveness of the NP roughness below leading to a higher tilt angle. When there is no second polymer layer, the fluorosurfactant is unable to attach to the coating as effectively (physisorption instead of electrostatic interaction) and hence the hexadecane tilt angle increases.

Oil repellency of the layer-by-layer composite coatings was further investigated using a series of straight-chain alkane oils and the data is presented in Table 2 and Fig. 4b. The coating was found to remain superoleophobic for tetradecane, dodecane, decane, and octane, with only slight increases in tilt angles for the latter two. This increase is due to the lower surface tensions of decane and octane and potentially the decrease in hydrocarbon chain length enabling better penetration of these oils through the fluorosurfactant layer.

### Wear resistance of coated surface

**Figure 5:**
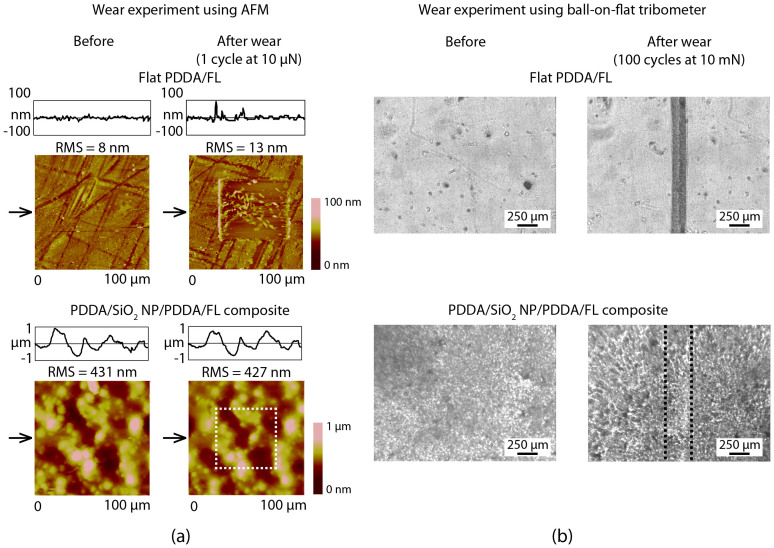
(a) Surface height maps and sample surface profiles (locations indicated by arrows) before and after AFM wear experiment with 15 μm radius borosilicate ball at a load of 10 μN for flat and optimized layer-by-layer composite coatings. RMS roughness values for surface profiles are displayed, and (b) optical micrographs before and after wear experiments using ball-on-flat tribometer at 10 mN for flat and layer-by-layer composite coatings.

The mechanical durability of the coatings was investigated through the use of AFM and tribometer wear experiments and images are shown in Fig. 5. The AFM images show a 100 × 100 μm^2^ scan area with the wear location (50 × 50 μm^2^) in the center of each image. For the soft PDDA/FL coating (ca. 230 nm thick), there is significant wear with both AFM and tribometer experiments causing observable damage to the surface. In contrast, the layer-by-layer composite coating survived the wear experiments with no observable defects. This suggests that the harder SiO_2_ nanoparticle layer (underneath ca. 80 nm thick PDDA/FL layers) helps improve the durability of the coating, while the PDDA binder layers help anchor the particles to the glass substrate.

**Figure 6:**
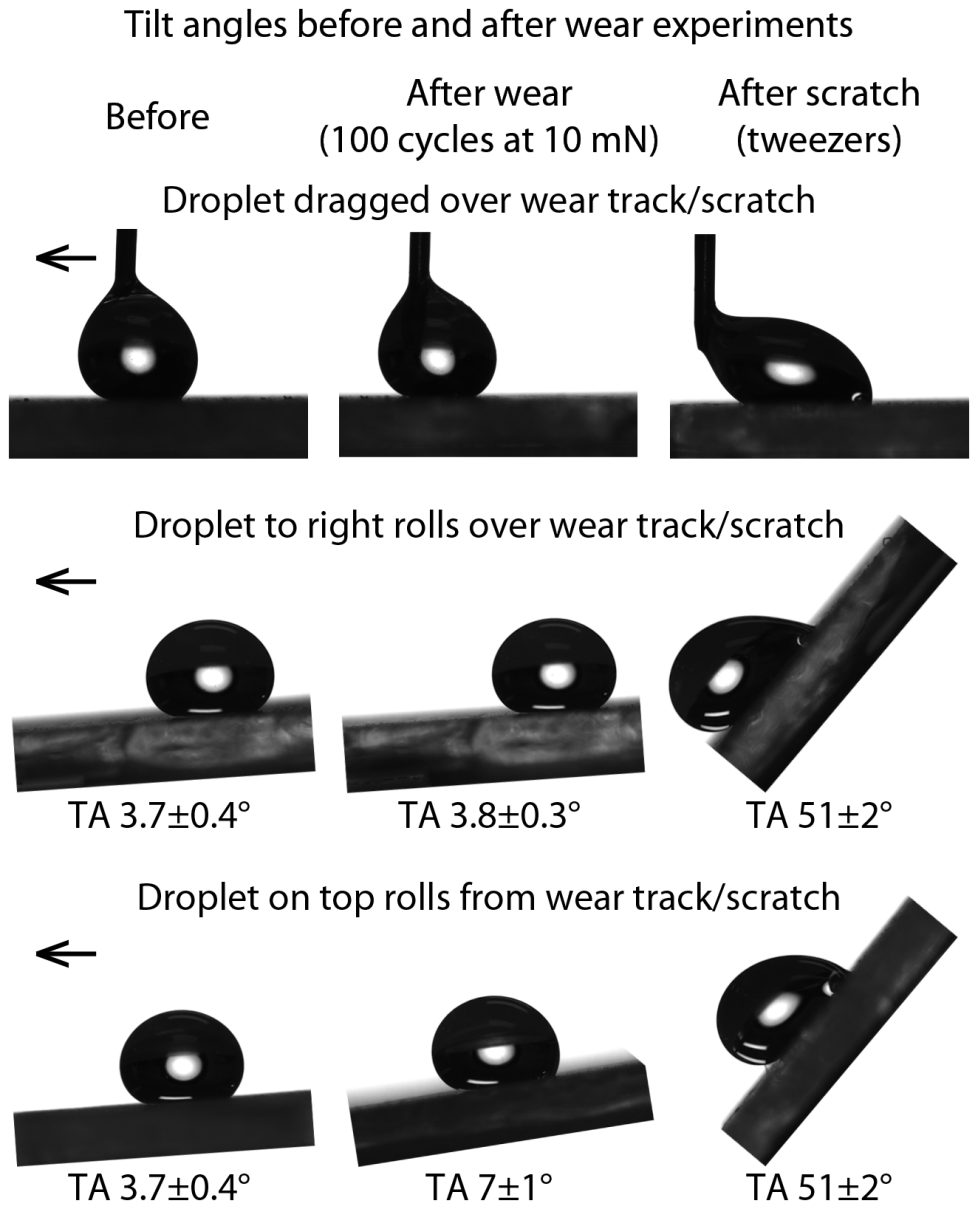
Images of hexadecane droplets on the optimized layer-by-layer composite coating before and after wear and scratching. Droplets were dragged or tilted across wear track (centered) in direction of arrows. For the worn samples (center column), droplets either rolled over the wear track as normal at 3.8 ± 0.3° tilt angle (when placed to the right of the defect) or rolled from the wear track after tilting the sample 7 ± 1° (when placed directly over the defect). Droplets on the scratched sample were pinned at the defect site until 51 ± 2° tilt angle regardless of starting location.

To further investigate the durability of the coating, hexadecane tilt angles were measured before and after tribometer wear experiments, Fig. 6. When droplets of hexadecane were tilted or dragged over the location of the wear experiment, the wear track was not found to impede the motion of the droplet. When deposited directly onto the wear track, there was a small increase in tilt angle before the droplet rolls away. This suggests that any damage caused to the surface is minimal and only leads to an increase in pinning for droplets deposited directly over the wear location. In contrast, coatings deliberately destroyed with tweezers resulted in a large increase in tilt angles with the hexadecane droplets pinning when deposited directly on the scratch or when tilted to roll over it.

### Anti-smudge property of coated samples

**Figure 7:**
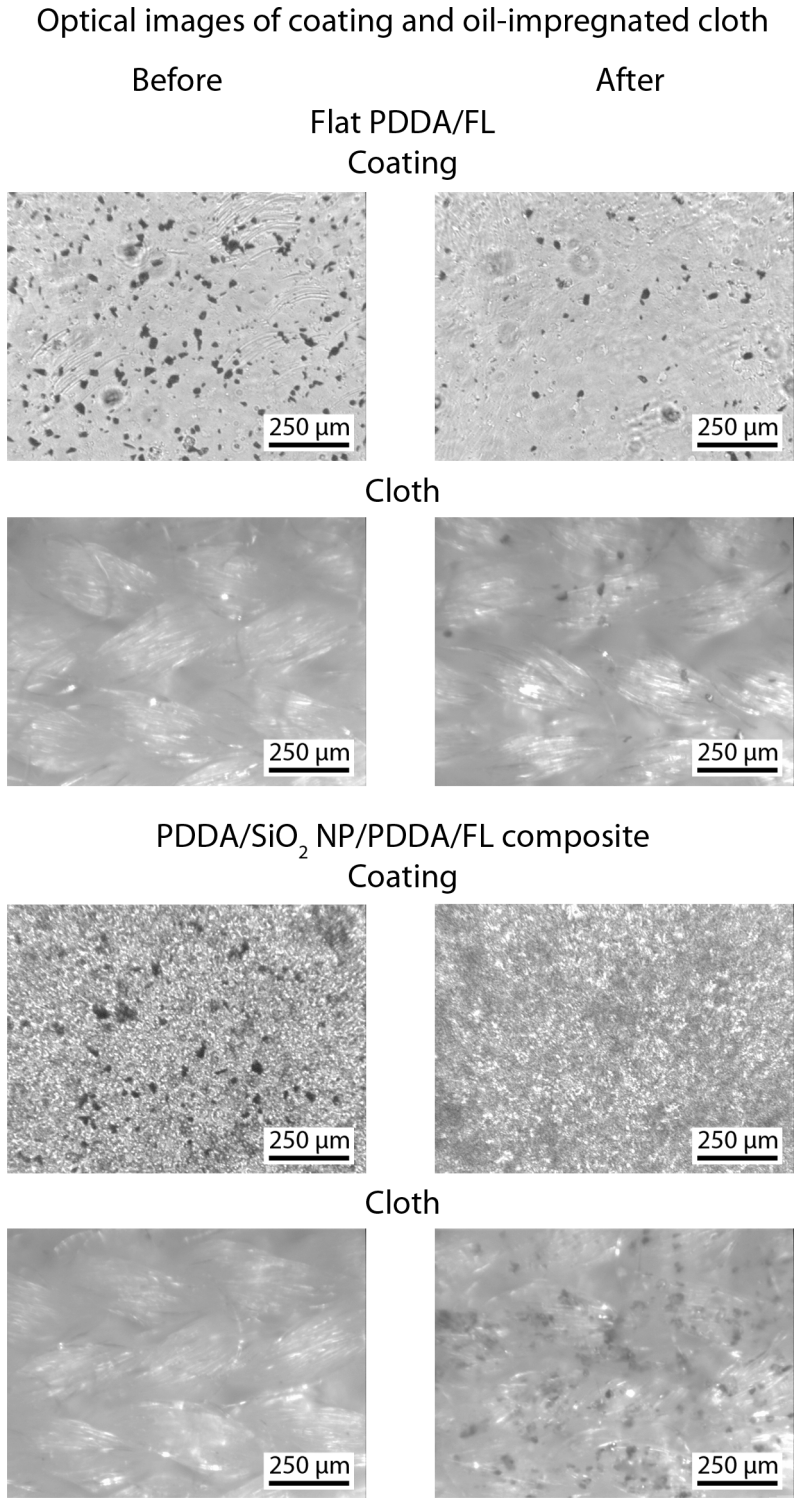
Optical micrographs of contaminated coatings and oil-impregnated microfiber cloth before and after smudge test on flat and optimized layer-by-layer composite coatings. Dark spots on coatings and cloth indicate silicon carbide particle contaminants.

To examine the anti-smudge properties, the coatings were contaminated with silicon carbide particles, Fig. 7. A hexadecane-soaked cloth was then used to wipe the surface. On the flat PDDA/FL coating this resulted in an incomplete removal of the particles with the surface remaining contaminated. For the layer-by-layer composite coating, the particles were transferred to the cloth with no observable particles remaining on the coating.

### Transparency of coated samples

**Figure 8:**
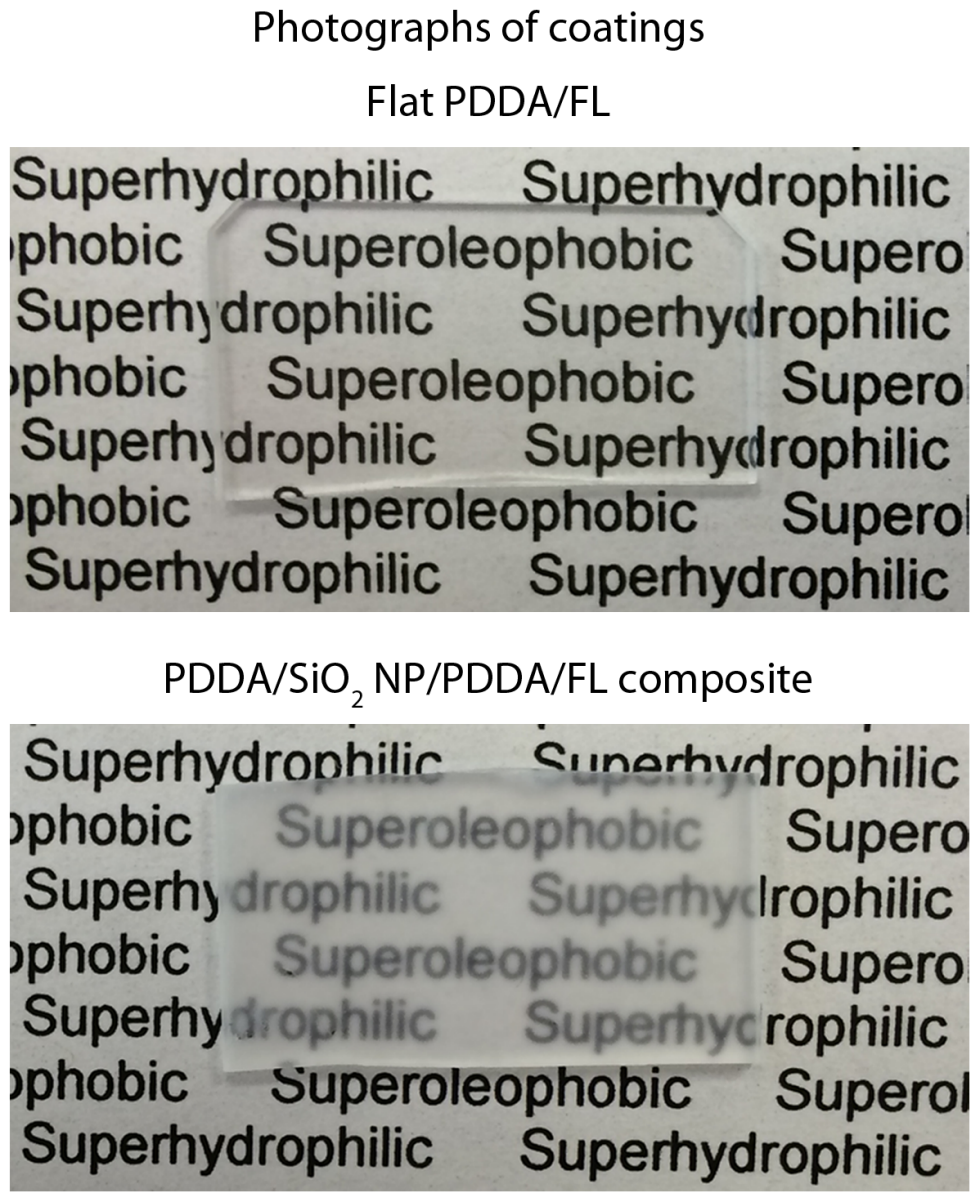
Photographs of flat and optimized layer-by-layer composite coatings. The flat coating appears transparent suggesting any reduction in transparency for the composite coating is due to the SiO_2_ nanoparticle layer.

Many applications of self-cleaning, anti-smudge surfaces rely on the transparency of the coating. When placed behind the layer-by-layer composite coating sample, text remains legible, suggesting that the coating displays characteristics of transparency, Fig. 8. The flat PDDA/FL coating exhibited similar transparency to bare glass suggesting that any decrease in transparency for the composite coating is due to the NP layer. Further improvement in transparency, potentially by decreasing the thickness of the NP layer, will be investigated in the future.

### Oil-water separation ability of coated samples

**Figure 9:**
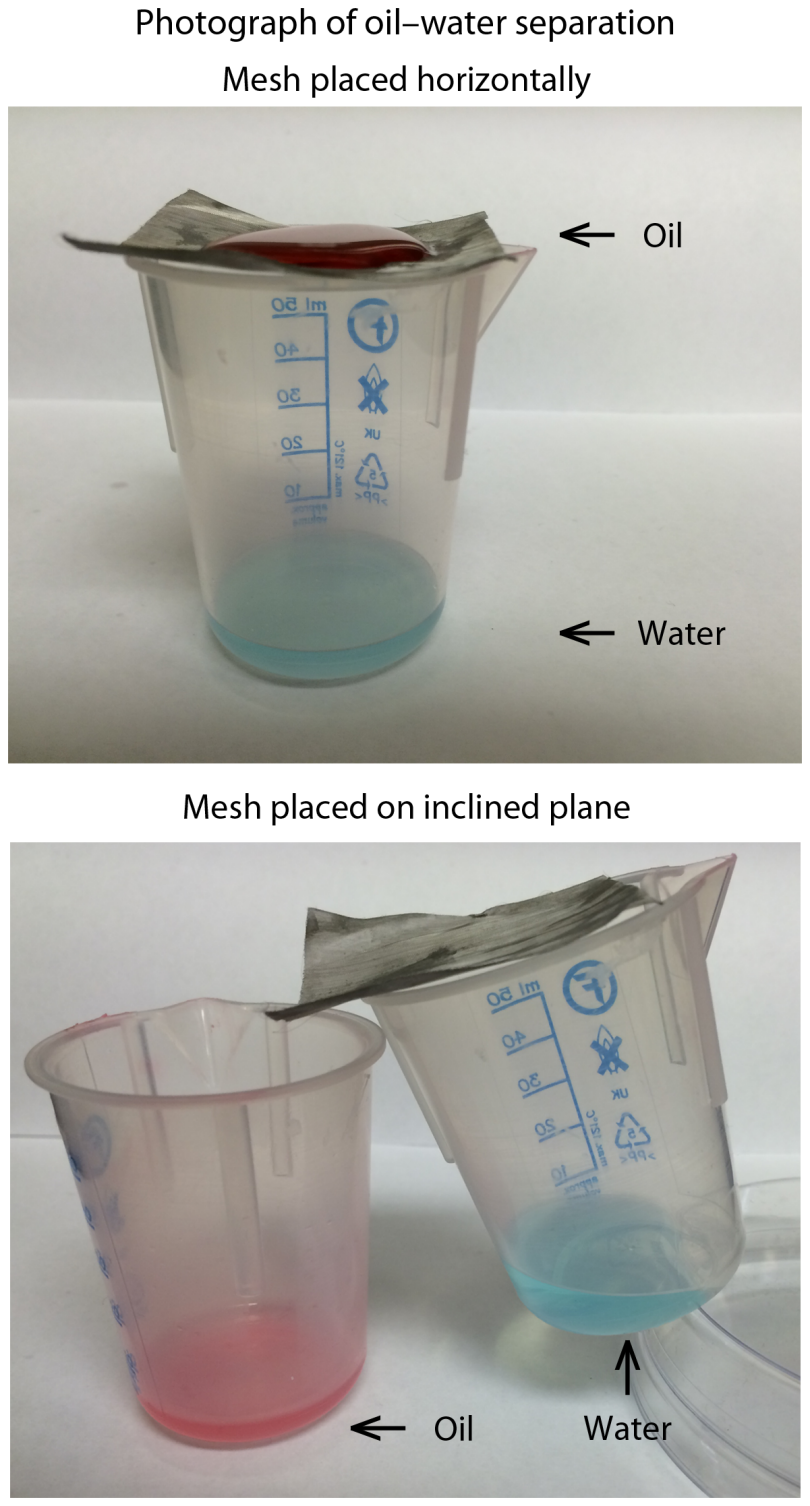
Photograph of optimized layer-by-layer composite coated stainless steel mesh acting as an oil-water separator. The superoleophobic/superhydrophilic nature of the coating results in water passing through the mesh while the hexadecane oil remains on the top surface of the horizontal mesh and can be easily rolled off. Alternatively the mesh can be placed on an inclined plane and oil and water collected simultaneously in separate beakers. Oil and water dyes used to enhance contrast.

The superoleophobic/superhydrophilic nature of the layer-by-layer composite coating enables its use as an oil-water separator. An agitated oil-water mixture was poured onto a coated mesh suspended over a beaker, Fig. 9. The water component of the mixture passed through the mesh while the oil component remained on top. The oil component could be easily removed by tilting with no contamination of the mesh. This is in contrast to typical oleophilic/hydrophobic oil-water separators, which can become fouled by oils requiring cleaning or replacement. Placing the mesh on an inclined plane resulted in the simultaneous collection of oil and water in two separate beakers.

These proof of concept experiments demonstrate that the layer-by-layer composite coating could find use in oil-water separation applications, however further work is required to determine their full effectiveness and suitability in real world applications.

## Conclusions

A durable, superoleophobic coating has been fabricated through the use of a layer-by-layer spray deposition technique. A polyelectrolyte-fluorosurfactant complex provides oleophobicity while remaining hydrophilic. Combined with SiO_2_ nanoparticles, the resulting coating is superoleophobic with hexadecane contact angles exceeding 155° and tilt angles of less than 4° due to the droplets being in the Cassie-Baxter wetting regime. The coating is also superhydrophilic with water contact angles of less than 5° due to water droplets being in the Wenzel regime. This combination of superoleophobicity and superhydophilicity is due to the fluorosurfactant containing a low surface tension fluorinated tail and a high surface tension head group. Large oil molecules are trapped at the fluorinated interface while smaller water molecules can more easily penetrate down to the hydrophilic region where the surfactant head group complexes with the polyelectrolyte layer.

The coating is found to be mechanically durable with micro- and macrowear experiments not causing any noticeable damage. After wear tests, hexadecane droplets were still found to roll off from the wear location suggesting any damage is minimal. The coatings were found to be anti-smudge with a hexadecane-soaked cloth removing all contaminants from the surface.

These surfaces were also found to display characteristics of transparency with text remaining visible through the coating. Layer-by-layer coatings are typically much thinner than reported here and it is believed that the current technique can be further optimized to reduce the thickness of the coating, thereby improving transparency.

The coating could find use in anti-fouling, self-cleaning, anti-smudge and low-drag applications, similarly to superoleophobic/superhydrophobic coatings. In particular, it could be useful in anti-biofouling, where superoleophobicity, superhydrophilicity and nanostructuring all contribute to reducing microorganism attachment.

Further, the fact that these coatings are superoleophobic/superhydrophilic means they could also be used for anti-fog and oil-water separation applications. When applied to a porous substrate, the coating was found to separate oil from water. Such coatings could help reduce the environmental impact of the gas, oil, metal, textile, and food-processing industries.

## Author Contributions

P.S.B. performed the experiments and analyzed the data. P.S.B. wrote the main text and P.S.B. and B.B. participated equally in planning, execution, and review of the manuscript.

## Figures and Tables

**Table 1:** Summary of layer-by-layer technique used in various applications

Application	Materials	Comments	Ref
Bioactive coatings	Proteins	Utilizes specific interaction between three different proteins to form layered film	49
	PEI, PSS and proteins	Utilizes specific interaction between two proteins to form layered film	50
Mineral thin films	PDDA and mineral	Technique results in ordered thin sheets of mineral	51
Organic dye thin films	Polylysine and dye molecules	Absorbance increases with increasing number of bilayers, technique leads to alignment of dye molecules	52
	Polyethyleneimine (PEI), poly(sodium 4-styrene sulfonate) (PSS), poly(diallyldimethylam-monium chloride) (PDDA) and dye molecules	Technique found to work for a variety of different dyes (different shape, size and charge distribution)	53
Conducting thin films	Polypyrrole (PPY) and PSS	Ultrathin, conducting films created using 10 bilayers	54
Chemical sensors	Self assembled monolayer (SAM) molecules	Reversible, selective binding makes detection of oligonucleotides possible	55
Microreactor	PEI and PSS	Glucose oxidase incorporated into layer-by-layer thin films and deposited on filter paper and used for reaction and product separation	56
Anti-reflective coating	PDDA, PSS and colloidal silica nanoparticles	Increasing number of bilayers (PSS/PDDA) improved transmittance of total film	57
	PAH, PSS and colloidal silica nanoparticles	50 nm particles on top of 100 nm particles led to improved anti-reflective properties	58
Superhydrophilicity	Poly(allylamine hydrochloride) (PAH), PSS and colloidal silica nanoparticles	16 bilayers (PAH/SiO_2_) result in a water contact angle of ca 0°. Surfaces used for anti-fogging	59
	PDDA, PSS and silica nanoparticles	Superhydrophilic and anti-reflective when 30 bilayers (PDDA/SiO_2_) deposited	60
	PSS, PDDA and Hierarchically mesoporous silica nanoparticles	Contact angles <5°, these transparent coatings could be further functionalized with silane to become superhydrophobic	38
	PDDA, PSS and silica nanoparticles	Used to coat microchannels for capillary-driven bioassays	39

**Table 2:** Comparison of various liquids deposited on flat (PDDA/FL) and layer-by-layer composite coatings

Liquid	Surface tension^61^ (mN m^−1^)	Flat PDDA/FL coating	Layer-by-layer composite coating	
		Contact angle (°)	Contact angle (°)	Tilt angle (°)
Octane	21.14	63 ± 1	153 ± 1	10 ± 1
Decane	23.37	68 ± 1	154 ± 1	7 ± 1
Dodecane	25.35	73 ± 1	158 ± 1	4 ± 1
Tetradecane	26.13	76 ± 1	157 ± 1	3.8 ± 0.3
Hexadecane	27.05	79 ± 1	157 ± 1	3.7 ± 0.4
Water	71.99	10 ± 2	<5	N/A
